# β-Cyanoalanine Synthases and Their Possible Role in Pierid Host Plant Adaptation †

**DOI:** 10.3390/insects8020062

**Published:** 2017-06-18

**Authors:** Anna-Maria Herfurth, Maike van Ohlen, Ute Wittstock

**Affiliations:** Institute of Pharmaceutical Biology, Technische Universität Braunschweig, Mendelssohnstrasse 1, D-38106 Braunschweig, Germany; a.herfurth@tu-bs.de (A.-M.-H.); m.vanohlen@hotmail.com (M.O.)

**Keywords:** glucosinolates, cyanide detoxification, Pieridae, β-cyanoalanine synthase, coevolution

## Abstract

Cyanide is generated in larvae of the glucosinolate-specialist *Pieris rapae* (Lepidoptera:Pieridae) upon ingestion of plant material containing phenylalanine-derived glucosinolates as chemical defenses. As these glucosinolates were widespread within ancient Brassicales, the ability to detoxify cyanide may therefore have been essential for the host plant shift of Pierid species from Fabales to Brassicales species giving rise to the Pierinae subfamily. Previous research identified β-cyanoalanine and thiocyanate as products of cyanide detoxification in *P. rapae* larvae as well as three cDNAs encoding the β-cyanoalanine synthases PrBSAS1-PrBSAS3. Here, we analyzed a total of eight species of four lepidopteran families to test if their cyanide detoxification capacity correlates with their feeding specialization. We detected β-cyanoalanine synthase activity in gut protein extracts of all six species tested, which included Pierid species with glucosinolate-containing host plants, Pierids with other hosts, and other Lepidoptera with varying food specialization. Rhodanese activity was only scarcely detectable with the highest levels appearing in the two glucosinolate-feeding Pierids. We then amplified by polymerase chain reaction (PCR) 14 cDNAs encoding β-cyanoalanine synthases from seven species. Enzyme characterization and phylogenetic analysis indicated that lepidopterans are generally equipped with one PrBSAS2 homolog with high affinity for cyanide. A second β-cyanoalanine synthase which grouped with PrBSAS3 was restricted to Pierid species, while a third variant (i.e., homologs of PrBSAS1), was only present in members of the Pierinae subfamily. These results are in agreement with the hypothesis that the host shift to Brassicales was associated with the requirement for a specialized cyanide detoxification machinery.

## 1. Introduction

**Figure 1:**
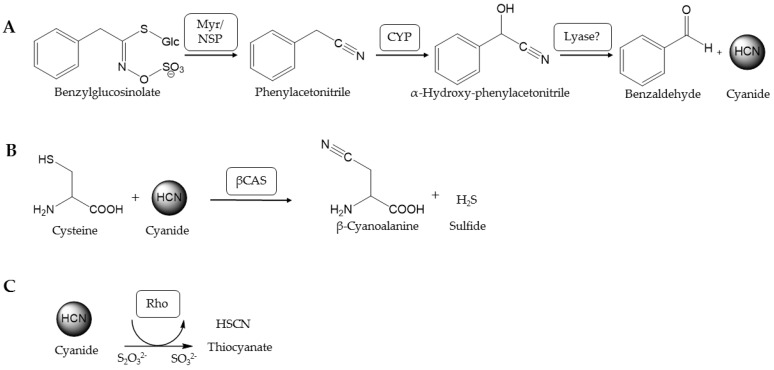
Cyanide formation and detoxification in *P. rapae*. (**A**) Formation of cyanide upon metabolism of benzylglucosinolate in *P. rapae* larvae. Aromatic glucosinolates are cleaved by plant myrosinases (Myr). In the presence of larval nitrile specifier protein (NSP), phenylacetonitrile is formed and further metabolized by gut cytochrome P450 enzymes (CYP) yielding instable α-hydroxynitriles which decompose to cyanide and aldehydes. (**B**) Cyanide detoxification catalyzed by β-cyanoalanine synthase (βCAS). (**C**) Cyanide detoxification catalyzed by rhodanese (Rho).

The glucosinolate-myrosinase system serves as an activated chemical defense in plants of the order Brassicales. Upon disruption of the cells, e.g., by herbivores, the glucosinolates, which are amino-acid derived thioglucosides, are hydrolyzed by endogenous thioglucosidases known as myrosinases [1] (Figure 1A). This yields products such as isothiocyanates, which are toxic to bacteria, fungi, nematodes and insects (reviewed in [2]). Despite this defense system, glucosinolate-containing plants are colonized by specialist and generalist insects [3,4,5,6]. Among the insects that feed exclusively on glucosinolate-containing plants are Pierid species of the Pierinae subfamily like *Pieris rapae* (Lepidoptera:Pieridae). Larvae of *Pieris rapae* possess a nitrile specifier protein (NSP) in their gut that redirects glucosinolate breakdown yielding simple nitriles instead of isothiocyanates [7] (Figure 1A). NSP activity has also been detected in a range of other glucosinolate-feeding Pierinae and is regarded as the key evolutionary innovation that allowed the host shift of Pierid butterflies to Brassicales about 80 million years ago [8]. Pierinae which performed a secondary host shift away from Brassicales appear to lack NSP activity [8]. While aliphatic nitriles formed upon glucosinolate metabolism in *P. rapae* larvae are excreted, aromatic nitriles are further metabolized [9,10,11]. Upon ingestion of benzylglucosinolate-containing plant material, the combined action of plant myrosinases and larval NSP results in formation of phenylacetonitrile whose further metabolism is associated with the release of equimolar amounts of cyanide [12] (Figure 1A). *P. rapae* larvae detoxify cyanide as β-cyanoalanine and thiocyanate indicating the involvement of β-cyanoalanine synthases and rhodaneses [12] (Figure 1B,C).

Three gut-expressed β-substituted alanine synthases (BSAS) with β-cyanoalanine synthase activity, PrBSAS1-PrBSAS3, have been cloned and characterized from *Pieris rapae* [13]. In phylogenetic trees, they are embedded in bacterial sequences together with uncharacterized proteins from other lepidopterans and with β-cyanoalanine synthase from the two-spotted spider mite, *Tetranychus urticae* (Trombidiformes: Tetranychidae), Tu-CAS [14]. The mite enzyme has been recruited by horizontal gene transfer from bacterial endosymbionts [14]. As a likely scenario, the presence of BSAS in lepidopteran genomes is due to an independent gene transfer event from bacteria to ancient lepidoptera [13,14]. Thus, arthropod BSAS are among the few known examples of horizontal gene transfer providing herbivores with tools to overcome plant chemical defenses [15].

**Figure 2:**
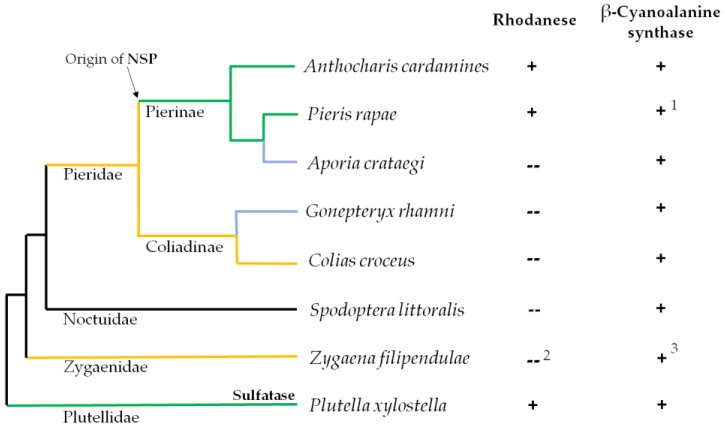
Presence of rhodanese and β-cyanoalanine synthase activities in lepidopteran species. Schematic representation of the phylogenetic relation between species based on [17]. Species were selected based on their feeding preferences and evolutionary history. Green lines: glucosinolate-feeding specialists; Yellow lines: species feeding on Fabaceae; Blue lines: species with secondary host shift to Rosaceae (*A. crataegi*) or Rhamnus-species (*G. rhamni*). Insect countermeasures against the glucosinolate-myrosinase system are indicated for glucosinolate-feeding specialists. Rhodanese (Rho) and β-cyanoalanine synthase (βCAS) activities were determined in extracts of different parts of the larvae (see Section 2.4 and Section 2.5 for details) unless reports on activity were available in the literature. + activity detected; -- no detectable activity. ^1^ according to [13]; ^2^ according to [18,19]; ^3^ according to R. Davis referred to in [18].

Phenylalanine-derived glucosinolates are among the evolutionarily oldest glucosinolates and were widespread in ancient Brassicales [16]. As the glucosinolate-feeding Pierinae diverged from mostly Fabales-feeding Coliadinae shortly after the appearance of the Brassicales [8], larvae of ancient Pierinae were likely confronted mostly with this type of glucosinolates and consequently with cyanide upon glucosinolate metabolism. The ability to colonize glucosinolate-containing plants may therefore have been associated with the ability to detoxify cyanide [12]. Here, we analyzed a number of Pierid and non-Pierid species with different feeding preferences and evolutionary histories (Figure 2) for β-cyanoalanine synthase and rhodanese activities. We then applied a polymerase chain reaction (PCR)-based strategy to clone cDNAs encoding β-cyanoalanine synthases from the seven species that had not been studied previously. This allowed us to compare the kinetic properties of the purified recombinant enzymes in relation to their phylogenetic relationships.

## 2. Materials and Methods

### 2.1. General

Protein concentrations were determined using the BCA Protein Assay Kit (Thermo Fisher Scientific, Waltham, MA, USA) with bovine serum albumine as a standard. PCR primers were Invitrogen Custom DNA Oligos (Thermo Fisher Scientific). Unless otherwise stated, PCR was carried out in a total volume of 50 µL DreamTaq buffer supplemented with 0.2 mM of each dNTP, 0.2 mM of each primer, 1 µL cDNA, and 0.25 µL DreamTaq Polymerase (Thermo Fisher Scientific) using PCR thermocyclers PeqStar (PEQLAB Biotechnology, Erlangen, Germany) and TProfessional Gradient (Biometra, Göttingen, Germany) and the following temperature program: 95 °C for 5 min, 35 cycles of 95 °C for 45 s, appropriate annealing temperature for 1 min, and 72 °C for 1 min, final incubation at 72 °C for 10 min. Sequencing was done at Eurofins MWG Operon (Ebersberg, Germany). β-Cyanoalanine was purchased from Sigma.

### 2.2. Larvae

All larvae were kept in a controlled environment chamber at 25 °C and 60% relative humidity with a photoperiod of 16 h with their respective host plants grown in or collected near the Institute of Pharmaceutical Biology. *P. rapae* larvae were from a colony maintained on *Brassica oleracea* ssp. *oleracea*, cv. Rosella (Brassicaceae) [13]. Larvae of *A. cardamines* were collected in Braunschweig, Germany, and kept on *Alliaria petiolata* (Brassicaceae) until dissection. Larvae of *A. crataegi* were obtained from M. Wasilewski, Białystok, Poland, and kept on *Sorbus aucuparia* (Rosaceae). Larvae of *G. rhamni* were collected in Braunschweig, Germany, and kept on *Frangula alnus* (Rhamnaceae). Larvae of *C. croceus* were obtained from G. Gęca, Będzin, Poland, and kept on *Lotus corniculatus* and *Medicago sativa* (Fabaceae). Larvae of *P. xylostella* were obtained from D. G. Heckel, Max Planck Institute for Chemical Ecology, Jena, Germany, and kept on Brussels sprouts (*Brassica oleracea* ssp. *oleracea*, cv. Rosella). Larvae of *Z. filipendulae* were obtained from M. Zagrobelny, University of Copenhagen, Denmark. Larvae of *S. littoralis* were obtained from V. Jeschke, Max Planck Institute for Chemical Ecology, Jena, Germany, and kept on an artificial bean diet [20,21].

### 2.3. Preparation of Larval Protein Extracts

*P. xylostella* larvae were extracted *in toto*. Larvae of the other species (late instar) were dissected into front part (“head”), gut tissue, gut content, and remaining parts (“integument”) in 50 mM Tris-HCl, pH 8.5, on ice. All tissues were rinsed with buffer and then ground in 1300 µL ice-cold 50 mM Tris-HCl, pH 8.5, using plastic pestles in 1.5 mL reaction tubes. After centrifugation at 20,000× *g* for 10 min, the supernatant was used as crude extract for enzyme assays.

### 2.4. Rhodanese Assay

Crude protein extracts (125 µL, 50 µg total protein) were incubated with 250 µL 125 mM KCN and 250 µL 125 mM sodium thiosulfate (both in 100 mM Tris-HCl, pH 8.5) at 30 °C for 120 min [22,23]. The reaction was stopped by addition of 125 µL formaldehyde and 625 µL ferric nitrate reagent [22]. After centrifugation for 5 min at 1050× *g*, the absorbance of the supernatant was measured at 460 nm and used to determine the SCN–concentration based on calibration with 0–1500 µM KSCN in 100 mM Tris-HCl, pH 8.5.

### 2.5. β-Cyanoalanine Synthase Assay

Crude larval protein extract (500 µL, 250 µg total protein) or purified recombinant protein (500 µL, 0.2–12 µg) was mixed with 250 µL 25 mM KCN and 250 µL 25 mM cysteine (both in 100 mM Tris-HCl, pH 8.5) [24]. Assay mixtures with purified recombinant enzymes were supplemented with 20 µM pyridoxal-5′-phosphate. The assay mixture was incubated at 31 °C for 10–120 min. For colorimetric detection of sulfide, 250 µL 20 µM N,N-dimethyl-p-phenylendiamindihydrochloride (DPD) in 7.2 M HCl and 250 µL 30 mM FeCl_3_ in 1.2 M HCl were added. After incubation in the dark for 20 min and centrifugation at 22,000× *g*, the absorbance of the supernatant was determined at 650 nm against a blank (100 mM Tris-HCl, pH 8.5, with added detection reagents) and used to determine the sulfide concentration based on calibration with 0–100 mM Na_2_S [24]. To verify the formation of β-cyanoalanine, the enzymatic reaction was stopped by addition of 100 µL formic acid and centrifuged at 22,000× *g*. The supernatant was analyzed by high-performance liquid chromatography-mass spectrometry (HPLC-MS) using Multiple Reaction Monitoring (MRM) as described previously [13] with *m/z* 112.722 as the mother ion (Q1) and *m/z* 95.900 as the daughter ion (Q3) [12].

For determination of kinetic parameters, the protein amount was varied to ensure linearity within the incubation time (10 min, 30 min for GrBSAS2). The protein amounts used for the kinetic characterization were 0.25 µg AcaBSAS1, 2.5 µg AcaBSAS2 and AcaBSAS3, 0.2 µg AcrBSAS1, 2 µg AcrBSAS2, 5 µg AcrBSAS3, 1 µg ZfBSAS2 and PxBSAS2, 5µg GrBSAS2, 3 µg CcBSAS2, 12 µg CcBSAS3, 2.5 µg SlBSAS2a and 0.4 µg SlBSAS2b (see Table 1 for abbreviations). The kinetics of cysteine were measured in the presence of 6 mM KCN with varying amounts of cysteine, the kinetics of cyanide in the presence of 6 mM cysteine with varying amounts of cyanide. The means of three technical replicates were used for nonlinear fitting to the Michaelis-Menten equation using OriginPro 8. *K*_m_ and *V*_max_ were determined as means ± standard error (SE) of *n* = 3 independent expression experiments.

### 2.6. RNA Isolation and cDNA Cloning

Total RNA was isolated from about 500 µL of frozen and ground larval tissue using the lithium chloride method according to [25] and analyzed spectrophotometrically and by agarose gel electrophoresis. PCR with degenerate primers P6-P13 (Table S1) was conducted on cDNA synthesized from 5 µg gut RNA with 55Scriptase (Nippon Genetics, Düren, Germany) and Oligo(dT)_20_ primer according to the manufacturer’s instructions. PCR were set up in a total volume of 50 µL Dream Taq Buffer supplemented with 0.8 µM of each primer, 0.2 mM of each dNTP, 2 µL gut cDNA and 0.25 µL DreamTaq polymerase. A hotstart touchdown temperature protocol was used (16 cycles of 95 °C for 45 s, 63–48 °C (−1 °C per cycle) for 1 min, and 72 °C for 1 min, 20 cycles of 95 °C for 45 s, 47 °C for 1 min, and 72 °C for 1 min and a final incubation at 72 °C for 10 min). PCR products were cloned into pGEM-T Easy (Promega, Madison, WI, USA) according to the manufacturer’s instructions and sequenced. To obtain 3′-ends, cDNA obtained using anchor-(dT)_18_ primer (Table S1) was subjected to PCR with a gene-specific primer (P14–26, Table S1) and the anchor primer. The SMARTer RACE cDNA amplification kit (Clontech, Mountain View CA, USA) was applied to obtain cDNA 5’ ends using a gene-specific primer and the SMARTer IIa oligonucleotide according to the manufacturer’s instructions. This cDNA was subjected to PCR with a gene-specific primer and primer RACElong, in most cases followed by nested PCR with the RACEshort primer and another gene-specific primer (P27–50, Table S1). PCR products were cloned into pGEM-T Easy (Promega) according to the manufacturer’s protocol and sequenced. In case of *Z. filipendulae*, sequence information of two entries in a transcriptome database [26] was kindly provided by M. Zagrobelny, University of Copenhagen, Denmark. One of the entries represented 3’-coding and untranslated region, the other 5’-coding and untranslated region, but there was no overlap between the two entries. A gene specific primer matching the 3’-coding sequence (P46, Table S1) was used for 5’-RACE together with primer RACElong. This amplified a cDNA which was identical to the two database entries in the respective 5’- and 3’-regions, respectively, demonstrating that both entries represented one transcript. For all cDNAs, full-length sequences were verified by independent amplification of the entire sequence using gene specific primers at the very 3’ and 5’ ends (P51–76, Table S1). In case of *P. xylostella*, the cDNA fragment amplified by PCR with degenerate primers matched database entry Px003388 of the diamondback moth genome database (http://iae.fafu.edu.cn/DBM). The ORF was amplified from *P. xylostella* cDNA using gene-specific primers designed based on the database entry (P77–78, Table S1). GenBank accession numbers are given in Table 1.

### 2.7. Generation of Expression Constructs

To generate expression constructs for recombinant enzymes with an N-terminal Strep-tag, the ORFs were amplified by PCR with 1 µL cDNA, 0.4 µM of each primer (P79–106, Table S1), and 0.5 µL *Pfu*Turbo Cx Hotstart Polymerase (Agilent) in a total volume of 25 µL. The PCR products were USER-cloned [27] into pET52b(+) (Novagen) modified for USER cloning as described [28] and sequenced.

### 2.8. Heterologous Expression and Purification of Recombinant Proteins

*E. coli* BL21 (DE3) pLysS (Thermo Fisher Scientific) was transformed with the expression constructs or the expression vector without insert as a control. A starter culture was used to inoculate 100 mL of TB medium with 100 mg/L ampicillin and 34 mg/L chloramphenicol to an OD_600_ of 0.1–0.15. This culture was incubated at 18 °C and 220 rpm until the OD_600_ was 0.4–0.5. Protein expression was then induced by the addition of 1 mM IPTG. After another 15 h incubation at 18 °C and 220 rpm, cells were harvested and resuspended in 1.5 mL 100 mM Tris-HCl, pH 8.0, 150 mM NaCl, 1 mM EDTA per 1 g of cells. After sonication and centrifugation, the supernatant was loaded onto Strep Tactin sepharose resin (IBA, Göttingen, Germany) for enzyme purification according to the manufacturer’s instructions. The elution fractions were analyzed by sodium dodecyl sulfate-polyacrylamide gel electrophoresis (SDS-PAGE) and the fractions with the highest amounts of purified recombinant proteins were pooled and used in enzyme assays to determine kinetics or frozen at −20 °C to be used later in enzyme assays for HPLC-MS (MRM) detection of β-cyanoalanine.

### 2.9. Statistics and Phylogenetic Analysis

Statistics on results of larval extract assays were done with OriginPro 8.0.63.988 SR6. Characterized BSAS were subjected to phylogenetic analysis together with all uncharacterized lepidopteran homologs identified through BLASTp searches against non-redundant protein sequences at NCBI using PrBSAS1 as query. Phylogenetic analysis was performed using MEGA7 after aligning the sequences with the MUSCLE algorithm [29,30,31]. The Maximum Likelihood tree was built with 1000 bootstrap replications and default settings (Jones-Taylor-Thornton (JTT) model with uniform rates).

## 3. Results

### 3.1. β-Cyanoalanine Synthase and Rhodanese Activity in Larvae

**Figure 3:**
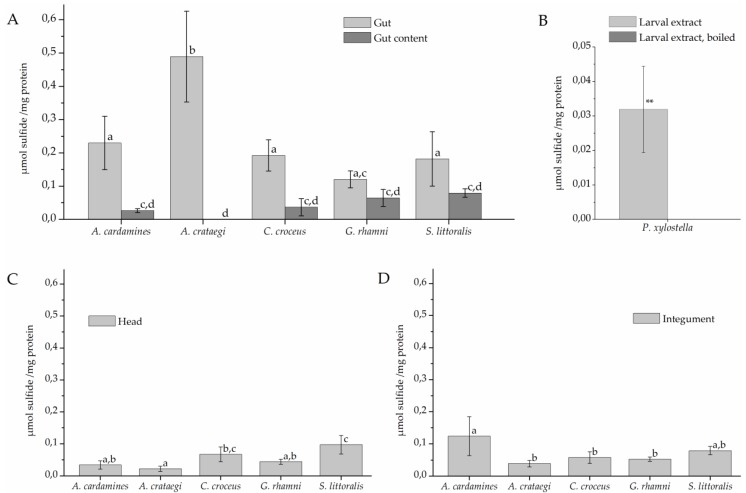
β-Cyanoalanine synthase activity in lepidopteran larvae. Protein extracts were prepared from larval gut tissue (**A**), gut content (**A**), head (**C**), and integument (**D**) or from whole larvae (**B**) and incubated with 6.25 mM cyanide and 6.25 mM cysteine for 120 min. Sulfide formation was measured colorimetrically. Shown are means ± standard deviation (SD) (*n* = 4–5 larvae). Different letters above columns in A, C, and D indicate a significant difference (*p* < 0.05, ANOVA with Tukey’s test). The mean background level (± SD, *n* = 7) obtained with heat-denatured gut extract was 0.009 ± 0.013 µmol/mg across all species used in A, C, and D. In B, asteriscs indicate a significant difference between larval extract and boiled control (**, *p* < 0.01, *t*-test).

To test if the larvae possess β-cyanoalanine synthase activity and, if so, where this activity is localized, larvae were dissected and protein extracts of different parts were incubated with cyanide and cysteine for 120 min (Figure 3). Sulfide was generated in assays with gut extracts of all investigated species at mean levels between 0.1 and 0.5 µmol/mg while only background levels (<0.03 µmol/mg) were obtained with heat-denatured gut extracts (Figure 3A). Levels measured in assays with gut extract were significantly higher than levels obtained in assays with gut content which were generally low (<0.1 µmol/mg). As the only exception, gut extracts of *G. rhamni* produced, on average, more sulfide than extracts of gut content, but this difference was not significant in most comparisons (Figure 3A). Assays with head extracts of some species also produced sulfide, but at low levels (Figure 3C). In addition, sulfide was formed at intermediate levels in incubations with *A. cardamines* integument extracts (0.12 µmol/mg), but at levels <0.1 µmol/mg in integument extracts of the other species (Figure 3D). In assays with extracts of whole *P. xylostella* larvae, significantly more sulfide was produced than in assays with boiled larval extract (Figure 3B).

**Figure 4:**
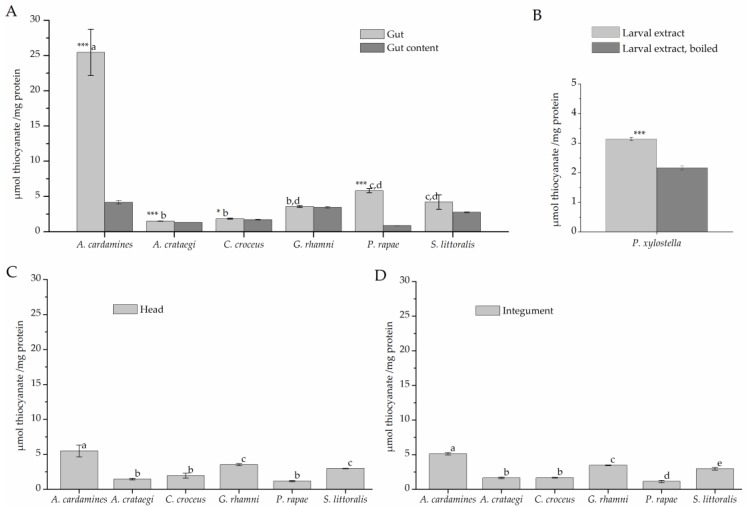
Rhodanese activity in lepidopteran larvae. Protein extracts were prepared from larval gut tissue (**A**), gut content (**A**), head (**C**), and integument (**D**) or from whole larvae (**B**) and incubated with 50 mM cyanide and 50 mM thiosulfate for 120 min. Thiocyanate formation was measured colorimetrically. Shown are means ± SD (*n* = 3–5 larvae). Different letters above columns in A, C, and D indicate a significant difference (*p* < 0.05, ANOVA with Tukey’s test) in a comparison of one sample type (gut tissue, head, integument) across species. Asterisks in A indicate a significant difference between gut tissue and gut content within a species (*, *p* < 0.05; ***, *p* < 0.001, *t*-test). The mean background level (± SD, *n* = 12) obtained with heat-denatured gut extract was 4,1 ± 2.9 µmol/mg across all species used in A, C, and D. In B, asteriscs indicate a significant difference between larval extract and boiled control (***, *p* < 0.001, *t*-test).

To test for rhodanese activity, larval extracts were incubated with cyanide and thiosulfate for 120 min (Figure 4). The highest level of thiocyanate formation (about 25 µmol/mg) was observed in assays with *A. cardamines* gut extract. Gut extracts of all other species, except *P. rapae*, and extracts of gut content produced thiocyanate at <5 µmol/mg protein (Figure 4A), a level close to that obtained with heat-denatured gut extract. In case of *P. rapae*, a more detailed analysis showed that gut extract produced significantly more thiocyanate than *P. rapae* gut content and heat denatured *P. rapae* gut extract (*p* < 0.001, *n* = 5). Incubations with head and integument extracts resulted in thiocyanate formation close to background levels with highest levels measured for samples from *A. cardamines* (Figure 4C,D), likely due to contamination of the material with gut activity. Although thiocyanate levels measured in assays with *P. xylostella* larval extract were very low, they were significantly higher than those measured in assays with boiled larval extract (Figure 4B). Taken together, β-cyanoalanine synthase activity was detected in all species under investigation with highest levels in gut tissue, while considerable rhodanese activity was only present in few species, namely *A. cardamines*, *P. rapae*, and *P. xylostella*.

### 3.2. Cloning of β-Cyanoalanine Synthase cDNAs

In order to obtain cDNAs encoding β-cyanoalanine synthases of *A. cardamines*, *A. crataegi*, *G. rhamni*, *C. croceus*, *P. xylostella*, and *S. littoralis*, we applied a previously developed PCR strategy with four forward and four reverse degenerate primers [13]. Using gut cDNA as a template, products of the expected size were obtained for each species under investigation, cloned and sequenced. This showed that cDNA fragments with high sequence similarity to previously cloned β-cyanoalanine synthase cDNAs from *Pieris rapae* [13] had been amplified. The cDNA fragment obtained from *P. xylostella* corresponded to database entry Px003388 of the diamondback moth genome database. In case of *Z. filipendulae*, gene-specific primers were designed based on transcriptome data [26] and used to amplify cDNA fragments with similarity to β-cyanoalanine synthase cDNAs. cDNA ends were completed by 3′ and 5′RACE (or using gene-specific primers derived from the database entry in case of *P. xylostella*) and ORFs confirmed by independent amplification of the entire sequence in one PCR. Several cDNAs were obtained for most species and designated as putative *BSAS* cDNAs (Table 1). The *BSAS* cDNAs encode polypeptides of 322–335 amino acids with a molecular weight of 34.4–35.9 kDa (Table 1) and a predicted cytosolic localization based on TargetP [32]. The assignment of numbers (*BSAS1-BSAS3*) was based on amino acid sequence similarity to PrBSAS1-PrBSAS3 [13] (Table 1).

### 3.3. Characterization of the Cloned BSAS as β-Cyanoalanine Synthases

**Figure 5:**
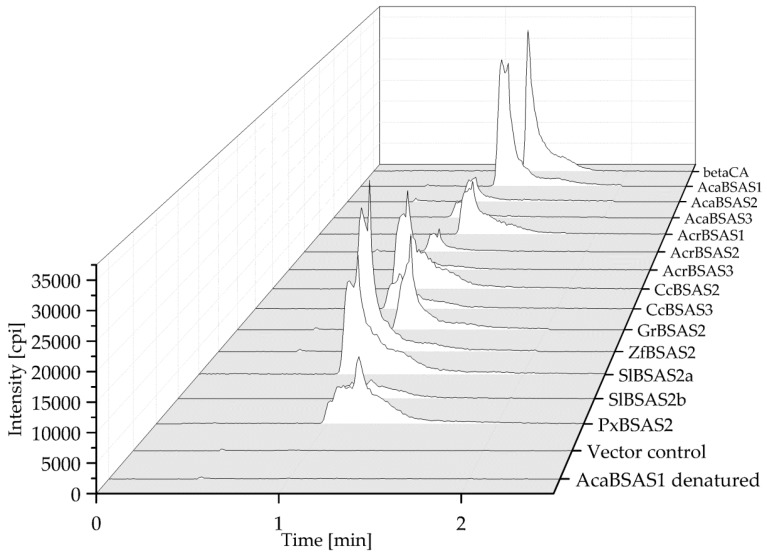
β-Cyanoalanine formation by lepidopteran BSAS. Purified recombinant AcaBSAS1, AcaBSAS2, AcaBSAS3, AcrBSAS1, AcrBAS2, AcrBSAS3, CcBSAS2, CcBSAS3, GrBSAS2, ZfBSAS2, SlBSAS2a, SlBSAS2b, PxBSAS2, heat denatured AcaBSAS1 or an equal volume of pooled elution fractions of the empty vector control were incubated with cysteine and cyanide in the presence of pyridoxal-5′-phosphate for 10 min. The reaction mixtures and a β-cyanoalanine standard (beta-CA) were analyzed by HPLC-MS/MRM. Shown are HPLC-MS/MRM traces depicting the *m/z* 112.7 to *m/z* 95.9 transition. Data for GrBSAS3 are shown with a different scale in Figure S2.

To test if the isolated cDNAs encode β-cyanoalanine synthases, the enzymes were expressed in *E. coli* in N-terminal fusion with a Strep-tag and purified via Strep-Tactin Sepharose (Figure S1). After incubation of the recombinant enzymes with cyanide and cysteine in the presence of pyridoxal-5′-phosphate for 10 min, reaction mixtures were analyzed for β-cyanoalanine by HPLC-MS/MRM. The transition expected for β-cyanoalanine (*m/z* 112.7–*m/z* 95.9) was observed in reaction mixtures with all enzymes tested, but not in reactions set up with heat-denatured enzyme or an equal volume of pooled elution fractions obtained from empty vector control (Figure 5 and Figure S2). Thus, the enzymes encoded by *AcaBSAS1-AcaBSAS3*, *AcrBSAS1-AcrBSAS3*, *GrBSAS2-GrBSAS3*, *CcBSAS2-CcBSAS3*, *SlBSAS2a-SlBSAS2b*, *ZfBSAS2* and *PxBSAS2* possess β-cyanoalanine synthase activity.

**Figure 6:**
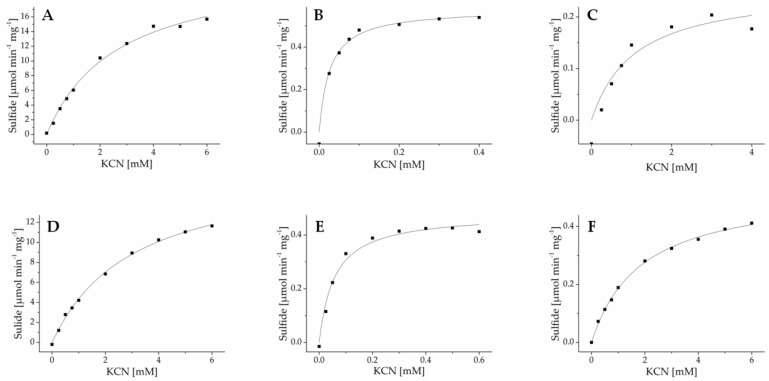
Kinetics of β-cyanoalanine synthases from Pierinae. AcaBSAS1 (**A**), AcaBSAS2 (**B**) and AcaBSAS3 (**C**) from *A. cardamines* and AcrBSAS1 (**D**), AcrBSAS2 (**E**) and AcrBSAS3 (**F**) from *A. crataegi* were incubated with 6 mM cysteine and varying cyanide concentrations in the presence of pyridoxal-5′-phosphate and sulfide formation determined colorimetrically. Shown are the results of one out of three independent experiments. Each data point represents the mean of three technical replicates. The curves were generated by nonlinear fitting to the Michaelis-Menten equation. The kinetics of BSAS from other species are shown in Figure S3.

To determine enzyme kinetics, purified recombinant enzymes were subjected to β-cyanoalanine synthase assays with varying substrate concentrations, and sulfide formation was determined in a colorimetric assay. GrBSAS3 could not be obtained in sufficient amounts for kinetic characterization. For all other enzymes, substrate dependence of activity followed Michaelis-Menten-kinetics (Figure 6 and Figure S3). *K*m values for cysteine were in the upper micromolar range for all newly identified enzymes and showed little variation among and within species (Table 2). *K*m values for cyanide varied by more than two orders of magnitude across all enzymes and by one to two orders of magnitude among isoenzymes from the same species (Table 2). For all species, one enzyme was found to have high affinity for cyanide (*K*m 20–40 µM). Typically, these enzymes had low turnover rates while the most efficient enzymes had *K*m values for cyanide in the low millimolar range. Taken together, this indicates that lepidopteran larvae may possess several β-cyanoalanine isoenzymes with different physiological functions.

### 3.4. Evolutionary Relationship Among β-Cyanoalanine Synthases

**Figure 7:**
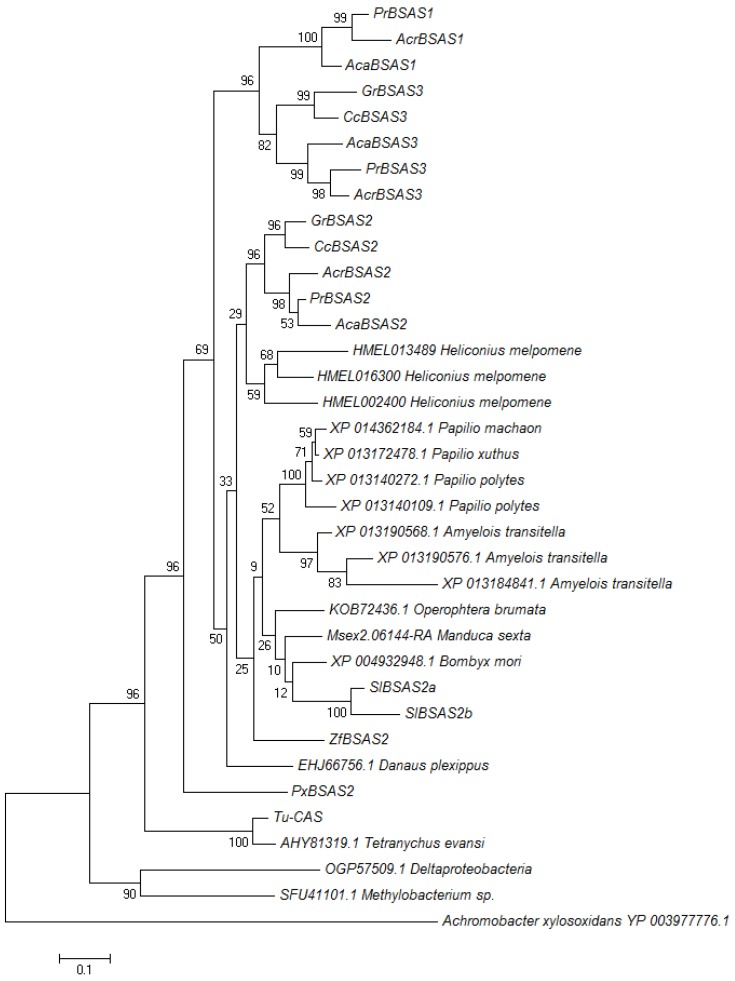
Phylogenetic relationship of characterized β-cyanoalanine synthases and uncharacterized homologs from lepidopteran species. Two enzymes from mites were also included. Uncharacterized bacterial homologs were used to root the tree. The Maximum Likelihood Tree was generated with 1000 bootstrap replications. Branch lengths refer to the number of substitutions per site, a scale bar is given below the tree.

When we subjected all characterized arthropod BSAS and uncharacterized lepidopteran homologs as well as bacterial sequences to phylogenetic analysis using the Maximum Likelihood algorithm, lepidopteran enzymes formed one branch separately from the mite β-cyanoalanine synthases (Figure 7). Lepidopteran enzymes clustered in three groups (Figure 7). While the first group contained PrBSAS2 and representatives from all analyzed species, the second group, which included PrBSAS3, was restricted to Pierid species. The third group, which included PrBSAS1, was formed exclusively by homologs from species of the Pierinae subfamily. Both homologs identified from *S. littoralis* belonged to the PrBSAS2-group. Uncharacterized homologs from Lepidoptera such as the the three homologs from *Heliconius melpomene* (Lepidoptera: Nymphalidae) also grouped together with members of the PrBSAS2-group, but not with PrBSAS1 or PrBSAS3 (Figure 7). Although we cannot exclude that more divergent BSAS genes exist in Lepidoptera, but have escaped our cloning strategy, the present data suggest that several lepidopteran species possess up to three BSAS homologs, but only in Pierids these have diverged to form separate groups.

## 4. Discussion

The present work was undertaken to test if glucosinolate-feeding Pierinae are equipped with specialized enzymes to detoxify cyanide released upon metabolism of glucosinolates inside the larval digestive system. As expected based on feeding experiments with *P. rapae* using isotopically labelled cyanide [12], we detected both rhodanese and β-cyanoalanine synthase activities in gut extracts of the two analyzed glucosinolate-feeding species of the Pierinae. Outside this group, however, we detected only scarce rhodanese activity. A previous study reported rhodanese activity to be widely distributed among insects of eight different orders, including Lepidoptera, although some species lacked rhodanese activity [18]. As the presence and extent of rhodanese activity did not correlate with the use of cyanogenic food plants, the study suggested that cyanide detoxification may not be the primary role of rhodaneses in insects [15]. The most common cyanogens in plants are the cyanogenic glycosides which are present in more than 130 plant families representing all major taxa of vascular plants [33]. Tissue disruption brings these chemical defenses together with co-occurring β-glycosidases leading to their hydrolysis with subsequent release of toxic cyanide, e.g., into the gut lumen of a herbivore. The lack of correlation observed in [18] could be due to the presence of physiological and behavioural avoidance mechanisms in certain insect species such as *Z. filipendulae* in addition to detoxification capabilities (reviewed in [34,35,36]) and does not exclude a role of rhodaneses in cyanide detoxification. Our results prompt us to hypothesize that formation of cyanide as a consequence of xenobiotic metabolism in glucosinolate-feeding Pierinae (rather than plant-mediated breakdown of cyanogens) may require additional measures against cyanide poisoning which may include rhodaneses expressed in gut tissue. Based on studies with *P. rapae*, cyanide release in glucosinolate-feeding Pierinae is expected to happen intracellularly after aromatic nitriles (derived from glucosinolates) have been converted to unstable α-hydroxynitriles, presumably by cytochrome P450 enzymes of the endoplasmic reticulum [12]. Immediate removal of cyanide by detoxification enzymes such as rhodaneses is likely to be essential to prevent fatal poisoning in these specialists whose larvae may produce several µg of cyanide per hour through benzylglucosinolate metabolism [12].

Besides rhodaneses, β-cyanoalanine synthases are likely to be involved in cyanide detoxification in herbivores, and the first arthropod β-cyanoalanine synthases have recently been identified and characterized [13,14]. In a screening of imagines of a broad range of lepidopteran species, β-cyanoalanine was detected in (but not restricted to) all species which also contained cyanogenic glycosides [37]. This supports a role of β-cyanoalanine synthases in the detoxification of cyanide, regardless of its generation from ingested or stored cyanogens. β-Cyanoalanine synthases are also involved in cyanide detoxification in plants and bacteria (reviewed in [38]). In the present study, extracts of isolated and thoroughly rinsed larval gut possessed high β-cyanoalanine synthase activities, in contrast to extracts of gut content. The ingested plant material is therefore an unlikely source of the activity detected in gut tissue. Although we cannot exclude that gut tissue contained intracellular or extracellular bacterial symbionts, previous analyses support the existence of gut expressed *BSAS* genes in mite and lepidopteran genomes which are likely to account for β-cyanoalanine synthase activity in the gut [13,14]. In support of recruitment of these genes from bacterial symbionts to arthropod genomes, Tu-CAS from *T. urticae* and PrBSAS1-PrBSAS3 are located within bacterial sequences in phylogenetic trees, but the corresponding genes show signs of amelioration, i.e., loss of bacterial in favour of arthropod signatures [13,14]. Moreover, *Tu-CAS* has been demonstrated to be part of the mite genome [14]. As the β-cyanoalanine synthases identified here group together with Tu-CAS and PrBSAS1-PrBSAS3 in phylogenetic trees, it is reasonable to assume that they have the same evolutionary background and are responsible for the detected enzyme activity in the gut.

In the present study, we demonstrate that each of the seven analyzed lepidopteran species has one β-cyanoalanine synthase with high affinity for cyanide supporting a role in cyanide detoxification. The enzymes with high affinity for cyanide all belong to the PrBSAS2 group which also includes uncharacterized lepidopteran homologs identified in databases. This suggests that β-cyanoalanine synthases of the PrBSAS2 group provide lepidopterans with a basic protection against cyanide released upon ingestion of cyanogenic plant material or hydrolysis of stored cyanogens. Some lepidopterans possess several homologs within the PrBSAS2 group, maybe due to a high frequency of encountering cyanide-producing food plants. Notably, Heliconius species such as *H. melpomene* with three BSAS2 homologs encoded in the genome feed exclusively on cyanogenic plants, namely Passiflora species, and are able to sequester and de novo synthesize cyanogenic glycosides [39,40]. As a very surprising result, we found Pierid species to possess additional β-cyanoalanine synthases which have diverged from those of the PrBSAS2 group and possess different kinetic properties. Of these additional β-cyanoalanine synthases, those of the PrBSAS3 group are expressed in all Pierid species analyzed, while those of the PrBSAS1 group appear to be restricted to Pierinae. Based on their nucleotide sequence identity of 65–70% within a species and their deviating kinetic properties, we suppose that they are BSAS homologs that have evolved by gene duplication, although we cannot exclude the possibility that the sequences represent divergent alleles. The enzymes of the PrBSAS1 (Pierinae) group have an about 50–100 fold lower affinity for cyanide, but an about 30 fold higher turnover number than members of the PrBSAS2 (Lepidoptera) group. Therefore, we assume that, next to rhodaneses and BSAS2 homologs, enzymes of the PrBSAS1 group may also be able to prevent cyanide accumulation in cells of the gut tissue upon larval metabolism of glucosinolate-derived aromatic nitriles. The presence of two or three BSAS homologs with different kinetic properties in Pieridae would also be in agreement with the observation that horizontally transfered genes are often subject to gene duplication followed by sub-or neofunctionalization [15].

Among the members of the PrBSAS2 (Lepidoptera) group, the two characterized enzymes from glucosinolate-feeding Pierinae, PrBSAS2 (from *P. rapae*) and AcaBSAS2 (from *A. cardamines*), have different properties. PrBSAS2 has an about tenfold lower affinity to cyanide than the other characterized group members including AcaBSAS2 (Table 2) putting its possible role in cyanide detoxification into question. It is interesting to note that one of the preferred host plants for *A. cardamines* oviposition and subsequent larval feeding is garlic mustard, *Alliaria petiolata* (Brassicaceae) [41], which possesses a unique pathway of glucosinolate breakdown. Upon homogenization, this plant converts its major glucosinolate, allylglucosinolate, to the corresponding thiocyanate (among other products) from which it produces cyanide [42]. Thus, AcaBSAS2 may have been preserved to meet this challenge posed by the host plant. Pieris species have a much broader host range than *A. cardamines* and rarely encounter plant-produced cyanide. Their interaction with *A. petiolata* as a host plant depends on species, habitat, and plant chemistry [43,44,45]. *P. rapae* larvae can complete development on *A. petiolata* [46], but adults prefer cabbage over *A. petiolata* for oviposition [47]. Future research will have to show if suitability of *A. petiolata* as a host plant for Pieris species is associated with plant cyanide release and insect cyanide detoxification capabilities. While the present study focused on constitutively expressed detoxification enzymes, future studies should test if some of the identified enzymes or additional enzymes are induced upon cyanide exposure.

Unfortunately, kinetic constants for cyanide could not be obtained for the third homolog from *P. rapae*, PrBSAS3 (Table 2) [13]. The three representatives of the PrBSAS3 (Pieridae) group that we were able to characterize with respect to enzyme kinetics did not show a particularly high cyanide affinity or efficiency of cyanide conversion. Therefore, their possible roles remain elusive based on the present data. Besides β-cyanoalanine synthases, the BSAS family of enzymes also includes cysteine synthases (O-acetylserine (thiol) lyases) which convert O-acetylserine and hydrogen sulfide to cysteine and acetic acid [48]. Some bacterial, plant, and nematode BSAS possess dual β-cyanoalanine synthase and cysteine synthase activity [38,49,50]. Among the three BSAS of the nematode *Caenorhabditis elegans*, only CYSL-2 functions as β-cyanoalanine synthase in cyanide detoxification in vivo [49]. The physiological relevance of the other homologs, CYSL-1 and CYSL-3, which both possess cysteine synthase activity in vitro, is less clear [49]. A role in mediating hypoxia-induced gene expression through interaction with a regulatory protein has been proposed for CYSL-1 while CYSL-3 may regulate hydrogen sulfide signaling [49]. Future studies should test if the lepidopteran BSAS identified here possess additional activities such as cysteine synthase activity. This could prevent accumulation of toxic sulfide, one product of the β-cyanoalanine synthase-catalyzed reaction, by incorporating it into cysteine. Our attempts to detect cysteine synthase activity of the newly identified BSAS by complementation of a cysteine-auxotroph *E. coli* strain [50] have failed so far (data not shown) leaving this an open question. Based on amino acid sequence analysis, all β-cyanoalanine synthases that have been cloned from Arthropods are predicted to be localized in the cytosol. Experimental evidence is, however, missing. Given the existence of three distinct groups of β-cyanoalanine synthases in Pierinae, it would be very interesting to elucidate the precise distribution and localization of these enzymes within the larvae. Together with quantitative expression analysis and knock-out experiments, such studies may provide us with a better understanding of this enzyme family and its physiological roles in lepidopteran larvae.

## 5. Conclusions

Among the lepidopteran larvae analyzed, only the glucosinolate-feeding specialists possessed considerable rhodanese activity, while β-cyanoalanine synthase activity was present in gut extracts of all Pierid and non-Pierid species analyzed. A more detailed analysis of β-cyanoalanine synthases from a range of lepidopteran species indicated that lepidopterans are protected against cyanide by β-cyanoalanine synthases of the PrBSAS2 group, whose characterized members possess high cyanide affinity. Pierid species are equipped with additional β-cyanoalanine synthases (i.e., homologs of PrBSAS1 and PrBSAS3), with deviating kinetic properties. Among these, PrBSAS1 homologs are restricted to glucosinolate-feeding Pierinae and have low cyanide affinity, but may contribute to cyanide detoxification based on their high turnover numbers. Taken together, the results of our study support the hypothesis that Pierinae have been under selection pressure to maintain a broad repertoire of cyanide detoxification enzymes. Whether these enzymes have evolved to also fulfil additional functions remains an interesting subject for future investigations.

## Figures and Tables

**Table 1:** β-substituted alanine synthases (BSAS) identified from lepidopteran species. Listed are BSAS which have been identified in this study and characterized as β-cyanoalanine synthases. The length of the obtained cDNA sequence and the ORF is also given together with the GenBank accession number, and the calculated molecular weight (MW) of the encoded protein (http://web.expasy.org/compute_pi). Designation was based on highest amino acid sequence identity to either PrBSAS1, PrBSAS2 or PrBSAS3.

Species	Protein	cDNA/ORF (bp)	GenBank	MW (kDa)	Amino acid Sequence Identity (%)		
					PrBSAS1	PrBSAS2	PrBSAS3
*A. cardamines*	AcaBSAS1	1365/978	MF038037	35.1	89.5	70.5	71.1
	AcaBSAS2	1333/978	MF038036	34.9	64.6	91.4	72.0
	AcaBSAS3	1282/981	MF038035	34.8	71.7	73.2	85.3
*A. crataegi*	AcrBSAS1	1472/975	MF038034	35.0	88.9	64.2	67.3
	AcrBSAS2	1233/978	MF038033	34.7	65.2	90.5	72.3
	AcrBSAS3	1120/981	MF038032	34.9	72.3	72.3	89.0
*C. croceus*	CcBSAS2	1458/1005	MF038031	35.7	67.7	84.6	72.1
	CcBSAS3	1266/981	MF038030	34.9	73.9	72.6	76.4
*G. rhamni*	GrBSAS2	1532/978	MF038029	34.7	68.3	85.5	73.2
	GrBSAS3	1288/981	MF038028	34.9	69.9	70.2	74.2
*Z. filipendulae*	ZfBSAS2	1611/990	MF038027	35.4	66.2	75.7	66.9
*S. littoralis*	SlBSAS2a	1453/999	MF038026	35.6	67.7	75.7	68.7
	SlBSAS2b	1345/1008	MF038025	35.9	66.5	70.5	66.9
*P. xylostella*	PxBSAS2	-/969	MF038024	34.4	63.8	70.4	67.0

**Table 2:** Kinetic constants of the characterized β-cyanoalanine synthases. After incubation of the purified recombinant proteins with cysteine and cyanide in the presence of pyridoxal-5′-phosphate for 10 min, sulfide was quantified colorimetrically. Cyanide was used at 6 mM when cysteine concentrations were varied and cysteine was used at 6 mM when cyanide concentrations were varied. Given are the means ± SEM from *n* = 3 independent expression experiments. Pd, Pieridae; Pn, Pierinae; C, Coliadinae; Z, Zygaenidae; N, Noctuidae; P, Plutellidae; Data for PrBSAS1-PrBSAS3 [13] are listed for comparison.

	Family-Subfamily	Cysteine		KCN	
		V_max_ (µmol/min mg)	*K*_m_ [mM]	V_max_ (µmol/min mg)	*K*_m_ (mM)
**AcaBSAS1**	**Pd-Pn**	23.77 (±1.35)	0.41 (±0.01)	16.18 (±1.50)	1.95 (±0.37)
**AcaBSAS2**	**Pd-Pn**	1.76 (±0.21)	0.44 (±0.01)	0.58 (±0.07)	0.03 (±0.00)
**AcaBSAS3**	**Pd-Pn**	1.41 (±0.19)	0.96 (±0.04)	0.22 (±0.03)	0.61 (±0.03)
**AcrBSAS1**	**Pd-Pn**	13.96 (±1.81)	0.26 (±0.00)	11.80 (±0.21)	2.56 (±0.21)
**AcrBSAS2**	**Pd-Pn**	2.09 (±0.19)	0.58 (±0.04)	0.44 (±0.05)	0.04 (±0.00)
**AcrBSAS3**	**Pd-Pn**	1.12 (±0.14)	1.31 (±0.08)	0.47 (±0.09)	2.95 (±0.83)
**CcBSAS2**	**Pd-C**	1.04 (±0.03)	0.75 (±0.06)	0.21 (±0.04)	0.03 (±0.01)
**CcBSAS3**	**Pd-C**	0.43 (±0.05)	1.50 (±0.08)	0.12 (±0.01)	0.16 (±0.02)
**GrBSAS2**	**Pd-C**	0.24 (±0.01)	0.37 (±0.00)	0.05 (±0.01)	0.02 (±0.00)
**GrBSAS3**	**Pd-C**	n.d. ^2^	n.d. ^2^	n.d. ^2^	n.d. ^2^
**ZfBSAS2**	**Z**	2.99 (±0.48)	0.74 (±0.04)	0.79 (±0.11)	0.03 (±0.00)
**SlBSAS2a**	**N**	1.44 (±0.06)	0.60 (±0.01)	0.38 (±0.07)	0.03 (±0.00)
**SlBSAS2b**	**N**	11.59 (±0.87)	1.07 (±0.03)	6.41 (±0.80)	0.29 (±0.05)
**PxBSAS2**	**P**	3.72 (±0.46)	0.52 (±0.04)	1.12 (±0.09)	0.02 (±0.00)
**PrBSAS1 ^1^**	**Pd-Pn**	12.32 (±0.42)	0.42 (±0.04)	16.86 (±2.80)	7.78 (±1.54)
**PrBSAS2 ^1^**	**Pd-Pn**	2.81 (±0.30)	0.61 (±0.08)	0.71 (±0.05)	0.28 (±0.08)
**PrBSAS3 ^1^**	**Pd-Pn**	0.71 (±0.03)	1.27 (±0.10)	n.d. ^2^	n.d. ^2^

^1^ according to [13]; ^2^ n.d., not detectable (low protein yield).

## Supplementary Materials

The following are available online at http://www.mdpi.com/2075-4450/8/2/62/s1, Table S1: Degenerate oligonucleotides and primers used for 3′- and 5′-RACE, cloning of full-length-cDNAs and generation of expression constructs, Figure S1: SDS-PAGE analysis of purified recombinant BSAS. Figure S2: β-Cyanoalanine formation by GrBSAS3, Figure S3: Kinetics of β-cyanoalanine synthases from Lepidoptera.

## Abbreviations

The following abbreviations are used in this manuscript:

PCR	polymerase chain reaction
BSAS	β-substituted alanine synthases
NSP	nitrile-specifier protein
SDS-PAGE	sodium dodecyl sulfate-polyacrylamide gel electrophoresis
HPLC-MS	high performance liquid chromatography-mass spectrometry
MRM	multiple reaction monitoring
DPD	*N*,*N*-dimethyl-*p*-phenylendiamindihydrochloride
ORF	open reading frame
SD	standard deviation
