# Supplementary material for: β-Cyanoalanine Synthases and Their Possible Role in Pierid Host Plant Adaptation
Source: Insects. 2017 Jun 18;8(2):62. doi: 10.3390/insects8020062 (PMC5492076; doi:10.3390/insects8020062)
Supplement: Supplementary file 1 [file insects-08-00062-s001.pdf]

insects

MDPI

Supplementary Material

β-Cyanoalanine Synthases and Their Possible Role in Pierid Host Plant Adaptation

Anna-Maria Herfurth, Maike van Ohlen and Ute Wittstock

Table S1. Degenerate oligonucleotides and primers used for 3'- and 5'-RACE, cloning of full-length-cDNAs and generation of expression constructs.

Abbreviation	Name	Sequence (5'-3')
P1	anchor-oligo(dT)18	GGC CAC GCG TCG ACT AGT ACT TTT TTT TTT TTT TTT TT
P2	anchor	GGC CAC GCG TCG ACT AGT AC
P3	SMARTer IIA	AAG CAG TGG TAA CAA CGC AGA GTA CGC GGG
P4	RACElong	CTA ATA CGA CTC ACT ATA GGG CAA GCA GTG GTA TCA ACG CAG AGT
P5	RACEshort	CTA ATA CGA CTC ACT ATA GGG C
P6	for1_CYSb	GCN AAR TGY GAR TTY ATG AAY CC
P7	for2_CYSb	GGN AAY CAR GGI TGY GGN YTN GC
P8	for3_CYSb	GGN CCI GAR ATH TGG MGN CAR AC
P9	rev1_CYSb	GGN GTR TAY TTN ARN CCI GTR TC
P10	rev2_CYSb	ACR SAN ARI CCY TCY TTY TCN CC
P11	rev3_CYSb	GTY TGN CKC CAD ATY TCI GGN CC
P12	for1_CYSc	AYA TGG MDG CNC TNG GHG C
P13	rev1_CYSc	GCV CCR CTV GTR WAN CCM AC
P14	Acabsas1for1	GGT ATC AAA CCG GAA ATA GAG TGG
P15	Acabsas2for1	CAC AAC CCA TTC GAG GAT GTG AG
P16	Acabsas3for1	GCT GAT GTC GAA GAA GCC CTA GAG
P17	Acr1_for2	GAG TGG AGC TAA TGT GTG TGC G
P18	AcrBSAS1 for1	GGT AGT GAC CAT TCT GTT CGA CAC C
P19	Acr1for1	TCA ATT TCG ACA CAA TGG ACG
P20	AcrBSAS3 for1	CGT GCA TTA TAA AGA CCT GCT CGG
P21	GrBSAS1 for1	CAG GAG ACG CAT GGG TTG TTA CC
P22	PxCYS2 for1	GAA GCA ACG CGT TAT AAG AAC C
P23	CcrBSAS1 for1	GCA ACG AAG ACA ATT CAG CTG CG
P24	CcrBSAS2 for1	GGT ACA TTG CCT GAA GAC GCA TGG G
P25	SlBSAS1_for1	GAA GTT AGT CGG TGA GAA GGA AGG
P26	SlBSAS2_for1	GCT ATC AAA CTG TTG GAG TCA GG
P27	AcaBSAS1rev1	TGG CGG CTA AAG ATG AAG CGA TAT C
P28	AcaBSAS2rev1	CTC ACA TCC TCG AAT GGG TTG TG
P29	AcaBSAS2rev2	CGC TAC AAA AGC ATC GAC GTG G
P30	AcaBSAS3rev1	CTC TAG GGC TTC TTC GAC ATC AGC
P31	AcaBSAS3rev3	CCT AGA ACT GCG CAG ACG A
P32	Acr1_rev2	GGA ATT GAT TTA CGA AGA ATG CCC
P33	Acr1_rev2_nested	CCA TAA GAT CAG CAT TGG TCA CG
P34	Acr1rev3	GTT GAA CTG GTT AAC GTA GTA TGC G
P35	Acr1rev1	GGT TAC GGT AAG AGG ATG TCC
P36	Acr3_rev2	CCT GTG GTC TCA TAG TGT GCC
P37	Acr3_rev2_nested	GTC TGC AAA GGT GAC GTT TCC

P38	Gr1_rev2	CGC TGC CAT GTT GTC TTC G
P39	Gr1_rev2_nested	CTT CTA TAG GAA TTG ACA CGT CGG
P40	GrBSAS rev1	GGT TCT TAT AAC GCG TTG CTT C
P41	GrBSAS rev3	GCA AAC GTA CCC GCA GTA CC
P42	Ccr1_rev2	CAG CTG AAT TGT CTT CGT TGC
P43	CcrBSAS1 rev1	TGA GAG TTA GGG GAT GTC CTA GTA CTG C
P44	Ccr2_rev2	TCT CGA TGA ACC AGT AAA TGT GC
P45	Ccr2_rev2_nested	GCG TAA ACC TTC TTT CTC AGC
P46	Zf2rev1	TGG CCT CGT TGT TGA ATT GG
P47	SlBSAS1neu_rev1	CGT GAT TGG GCA TCC CG
P48	SlBSAS1neu_rev2	GTT GGC CTC GTT GTG AAA TTG
P49	SlBSAS2_rev	CTA CAG GTT CAC CAG GTT TCA GC
P50	SlBSAS2_rev_nested	CCT TGA TTG ATG CAC CAT GG
P51	Acabsas1full2for	CAG AGT ACG CGG GCT TCA TT
P52	Acabsas1full2rev	CTC TCC GAT TTA ATC TTT CAA CGC
P53	Acabsas2fullfor	CAG AAT AGA CAA GGC CTT AAG C
P54	Acabsas2fullrev	CCT ATG TAT ATT ATC ACT TTT GAT CC
P55	Acabsas3full2for	GGA GTG ACA ATT TAT ATC GTA GTG TAG
P56	Acabsas3fullrev	CTC TCT CCC TCT AAT TAA CAA AGC G
P57	Acr1_full_for	GGA GAT TTC GTT CGA ATT TCG
P58	Acr1_full_rev	GCA ACA CAT TTA GCT GAT CCG
P59	AcrCYSfull_for	CAG TGT TGA TTG ACA CCA GTA CAG
P60	AcrCYSfull_rev	GTA CTA AGA GAG GCA TAA TGT CTG AG
P61	Acr3_full_for	GGA TTC ATT TAG ACT GAT AAC ACA GC
P62	Acr3_full_rev	TAT ATA TCG GCC TTT AGC GTA CG
P63	Gr1_full_for2	CTG TAG ATT GAG TTC ACT GCT TAG G
P64	Gr1_full_rev2	AGC GAG AAA TGA ACT TGA AGC
P65	GrBSAS_full_for	CCG CTT TGC TTC AGT ATC AAT TGC
P66	GrBSAS_full_rev	CAC GTA CTC ATT TTG AGA GAC TTG C
P67	Ccr1_full_for	GAG GTG TAG GTA ATC GTG TAT TGG
P68	Ccr1_full_rev	ACG GAA TTG TTG CGA GTC G
P69	Ccr2_full_for2	GGT ATA ACT CAA TCA ACA CAT CAG C
P70	Ccr2_full_rev2	CAC GCT TAT CTC GAT GCC AC
P71	ZfCYSfull2for	CCT CAA GCG GTG AAA ACT TCC
P72	ZfCYSfull2rev	CAT GCG TGA CTC GGA CTA TGC
P73	SlBSAS1 full for neu	GGA GTA TCC AAC TGG TAT CTG C
P74	SlBSAS1 full rev	CCT CAG ATG TTA TCA ATC ACA ATT AAT CAC
P75	SlBSAS2 full for	CTC CAA GCT CAC CTC CAA CC
P76	SlBSAS2 full rev	GTT TAT TAC ACG ATA CAC ATA ACA AGC
P77	PxCYS for	ATG TCG ACC ATT GAC GGA GTT ATT G
P78	PxCYS rev	CTA GCA GTA CTT GAG GCC GGT GTC
P79	Acabsas1 for USER	GGC TTA AUA TGG GTC AGG CGT GC
P80	Acabsas1 rev USER	GGT TTA AUT CAC ATT AAA GAT TCT GGT ACA GG
P81	Acabsas2 for USER	GGC TTA AUA TGA CTA AGG AGA ACG G
P82	Acabsas2 rev USER	GGT TTA AUT TAT GTT AAG CTG TCT GGT AC
P83	Acabsas3 for USER	GGC TTA AUA TGT CTG ATT CAA ATG
P84	Acabsas3 rev USER	GGT TTA AUC CTC TAA TTA ACA AAG C
P85	AcrBSAS1 for USER	GGC TTA AUA TGG GTC AGA CGT GTG
P86	AcrBSAS1 rev USER	GGT TTA AUG ATC CGT TTT AAT AAA CTT GC

P87	AcrBSAS USER for	GGC TTA AUA TGG CTA AAG TGA ACG G
P88	AcrBSAS USER rev	GGT TTA AUT TAT ATT AAG CTT TCT GCT ACA GG
P89	AcrBSAS3 for USER	GGC TTA AUA TGA CTA ACG CAA TTA G
P90	AcrBSAS3 rev USER	GGT TTA AUC CTA AGT AAA ATT TAT TCG
P91	GrBSAS1 for USER	GGC TTA AUA TGG CTG GAC CAA GTC C
P92	GrBSAS1 rev USER	GGT TTA AUC TAG TTT ATT AAA GAT TCG CGA AC
P93	GrBSAS2 for USER	GGC TTA AUA TGG CGA AGG TGA AC
P94	GrBSAS2 rev USER	GGT TTA AUT TAT GTT AAA GAT TCT GGT ACT G
P95	Ccr1 for USER	GGC TTA AUA TGA CCT CAT CGA CTA A
P96	Ccr1 rev USER	GGT TTA AUC TAG TTT ATT AAT GAT TCT GG
P97	Ccr2 for USER	GGC TTA AUA TGG CAA AGG TGA ACG G
P98	Ccr2 rev USER neu	GGT TTA AUC TTG GTT AAT AAC TTT CTC G
P99	PxCYS for USER	GGC TTA AUA TGT CGA CCA TTG ACG GAG TTA TTG
P100	PxCYS rev USER	GGT TTA AUC TAG CAG TAC TTG AGG CCG GTG TC
P101	ZfBSAS for USER	GGC TTA AUA TGA GTC CGC CGG TAT TG
P102	ZfBSAS rev USER	GGT TTA AUC TAA TCG CTT AAT TCT ATC TCG TCT G
P103	SlBSAS1 USER for	GGC TTA AUA TGG CTC CCA TCG
P104	SlBSAS1 USER rev	GGT TTA AUT CAC TTG AAT AAC TCC TCA G
P105	SlBSAS2 USER for	GGC TTA AUA TGG CTC CCG TCG AGA AGA AC
P106	SlBSAS2 USER rev	GGT TTA AUT CAC TTC ATT AAT TCC TCT GGT ACT GGC

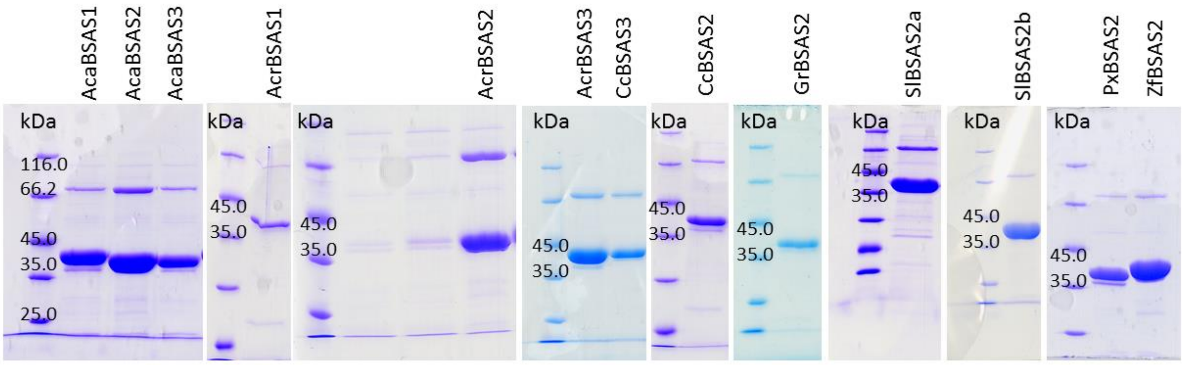

Figure S1. SDS-PAGE analysis of purified recombinant BSAS. Designation of proteins is as given in Table 2. Crude extracts of E. coli expressing the protein were purified by Streptactin-affinity chromatography. Pooled elution fractions were analyzed. Coomassie-stained gels are shown with protein ladder at the left side of each image. BSAS have a molecular weight of about 35 kDa.

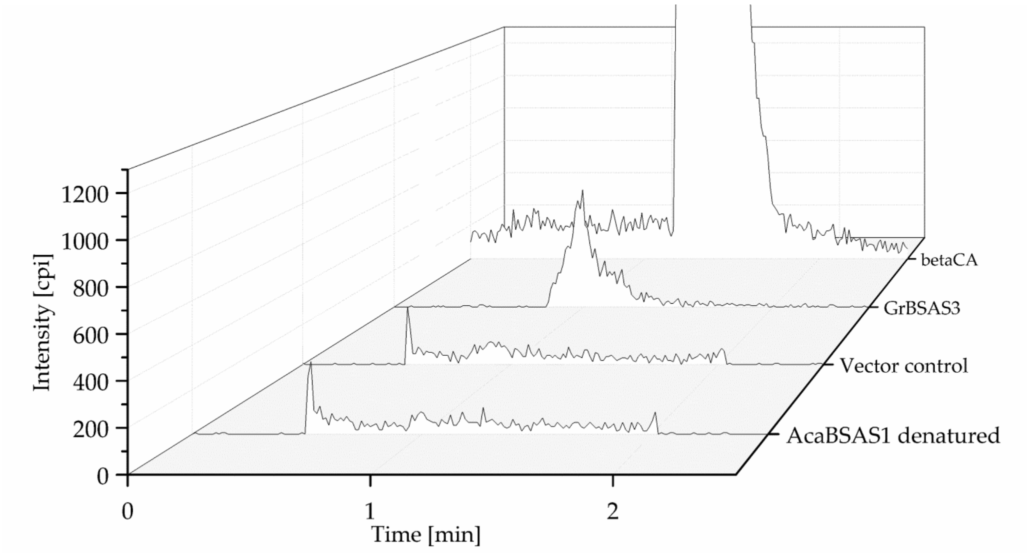

Figure S2. β-Cyanoalanine formation by GrBSAS3. Purified recombinant GrBSAS3, heat denatured AcaBSAS1 or an equal volume of pooled elution fractions of the empty vector control were incubated with cysteine and cyanide in the presence of pyridoxal-5'-phosphate for 10 min. The reaction mixtures and a β-cyanoalanine standard (beta-CA) were analyzed by HPLC-MS/MRM. Shown are HPLC-MS/MRM traces depicting the m/z 112.7 to m/z 95.9 transition.

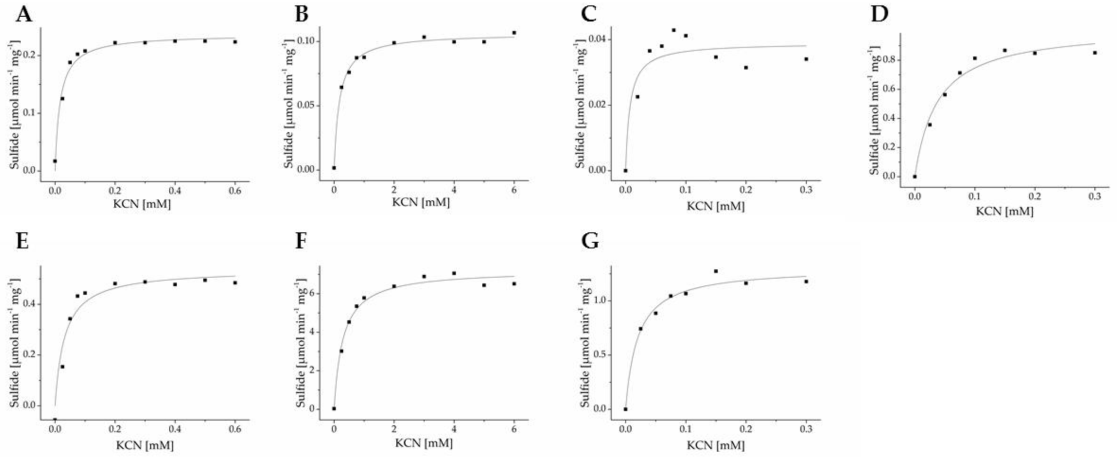

Figure S3. Kinetics of β-cyanoalanine synthases from Lepidoptera. CcBSAS2 (A) and CcBSAS3 (B) from *C. croceus*, GrBSAS2 (C) *from G. rhamni*, ZfBSAS2 (D) from *Z. filipendulae*, SlBSAS2a (E) and SlBSAS2b (F) from *S. littoralis* and PxBSAS2 (G) from *P. xylostella* were incubated with 6 mM cysteine and varying cyanide concentrations in the presence of pyridoxal-5′-phosphate and sulfide formation determined colorimetrically. Shown are the results of one out of three independent experiments. Each data point represents the mean of three technical replicates. The curves were generated by nonlinear fitting to the Michaelis-Menten equation.
